# Women’s Health Initiative Strong and Healthy Pragmatic Physical Activity Intervention Trial for Cardiovascular Disease Prevention: Design and Baseline Characteristics

**DOI:** 10.1093/gerona/glaa325

**Published:** 2021-01-12

**Authors:** Marcia L Stefanick, Abby C King, Sally Mackey, Lesley F Tinker, Mark A Hlatky, Michael J LaMonte, John Bellettiere, Joseph C Larson, Garnet Anderson, Charles L Kooperberg, Andrea Z LaCroix

**Affiliations:** 1 Department of Medicine, Stanford Prevention Research Center, Stanford University School of Medicine, California, USA; 2 Department of Epidemiology and Population Health, Stanford University School of Medicine, California, USA; 3 Public Health Sciences Division, Fred Hutchinson Cancer Research Center, Seattle, Washington, USA; 4 Department of Medicine, Primary Care and Outcomes Research, Stanford University School of Medicine, California, USA; 5 Department of Epidemiology and Environmental Health, School of Public Health and Health Professions, University at Buffalo–SUNY, New York, USA; 6 Department of Family and Preventive Medicine, Herbert Wertheim School of Public Health and Longevity Science, University of California San Diego, La Jolla, USA

**Keywords:** Cardiovascular, Falls, Physical activity, Physical function

## Abstract

**Background:**

National guidelines promote physical activity to prevent cardiovascular disease (CVD), yet no randomized controlled trial has tested whether physical activity reduces CVD.

**Methods:**

The Women’s Health Initiative (WHI) Strong and Healthy (*WHISH*) pragmatic trial used a randomized consent design to assign women for whom cardiovascular outcomes were available through WHI data collection (*N* = 18 985) or linkage to the Centers for Medicare and Medicaid Services (N30 346), to a physical activity intervention or “usual activity” comparison, stratified by ages 68–99 years (in tertiles), U.S. geographic region, and outcomes data source. Women assigned to the intervention could “opt out” after receiving initial physical activity materials. Intervention materials applied evidence-based behavioral science principles to promote current national recommendations for older Americans. The intervention was adapted to participant input regarding preferences, resources, barriers, and motivational drivers and was targeted for 3 categories of women at lower, middle, or higher levels of self-reported physical functioning and physical activity. Physical activity was assessed in both arms through annual questionnaires. The primary outcome is major cardiovascular events, specifically myocardial infarction, stroke, or CVD death; primary safety outcomes are hip fracture and non-CVD death. The trial is monitored annually by an independent Data Safety and Monitoring Board. Final analyses will be based on intention to treat in all randomized participants, regardless of intervention engagement.

**Results:**

The 49 331 randomized participants had a mean baseline age of 79.7 years; 84.3% were White, 9.2% Black, 3.3% Hispanic, 1.9% Asian/Pacific Islander, 0.3% Native American, and 1% were of unknown race/ethnicity. The mean baseline RAND-36 physical function score was 71.6 (± 25.2 *SD*). There were no differences between Intervention (*N* = 24 657) and Control (*N* = 24 674) at baseline for age, race/ethnicity, current smoking (2.5%), use of blood pressure or lipid-lowering medications, body mass index, physical function, physical activity, or prior CVD (10.1%).

**Conclusion:**

The *WHISH* trial is rigorously testing whether a physical activity intervention reduces major CV events in a large, diverse cohort of older women.

**Clinical Trials Registration Number:** NCT02425345

National guidelines promote physical activity and limiting sedentary behavior to prevent cardiovascular disease (CVD) and other chronic diseases in older adults (1,2). The U.S. population aged 65 years and older is projected to reach 95 million (23% of the population) by the year 2060, with persons aged 85 years and older reaching 19 million, the majority of whom will be women (3). Decades of evidence-based interventions have shown that increasing physical activity can improve levels of physical function and cardiovascular (CV) risk factors (4–7), but these trials have mostly enrolled subjects less than 75 years old, and have mostly tested supervised, clinic-based exercise programs. There have been no randomized controlled trials with physical activity as the sole intervention that tested the hypothesis that increasing physical activity and/or reducing sedentary time reduces the incidence of major CV events. While the LIFE Trial showed that a physical activity intervention modestly preserved mobility in older adults with low physical function (8), a pragmatic centralized physical activity intervention delivered to aging adults across the functional continuum has not yet been tested. We designed the *WHI Strong & Healthy (WHISH)* trial as a large-scale, randomized, controlled, pragmatic trial to fill this critical evidence gap. The *WHISH* trial is rigorously testing whether a physical activity intervention designed to deliver national physical activity recommendations for older adults reduces major CV events in a large, geographically diverse, multiethnic cohort of older women.

## Method

### Objectives and Outcomes

The primary hypothesis being tested is that a centralized, public health behavioral intervention designed to increase or maintain physical activity levels and reduce sedentary behavior will reduce the incidence of major CV clinical events, specifically myocardial infarction (MI), stroke, or CVD death, in older women. Secondary hypotheses are that the *WHISH* physical activity intervention will lower rates of venous thromboembolism, peripheral artery disease, and reduced physical functioning.

The primary safety aims are to evaluate whether the *WHISH* intervention increases risks of total clinical fracture, hip fracture, falls, or non-CVD mortality over ~8 years, compared with the usual follow-up (control). A secondary safety aim is to evaluate whether the physical activity intervention increases coronary artery revascularization (coronary artery bypass graft surgery or percutaneous coronary intervention).

The ultimate goal of the *WHISH* trial is to provide definitive evidence on the benefits and risks of this pragmatic physical activity intervention and, if efficacious, to disseminate an easily scalable intervention to improve the CV health of aging Americans.

### Randomized Consent Design

**Figure 1.**
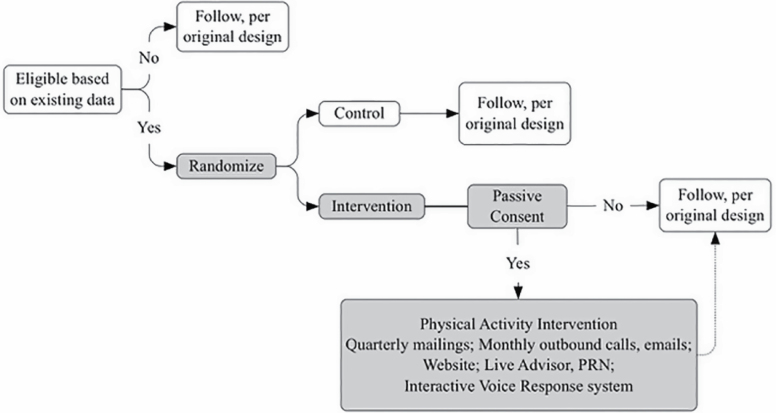
Randomized consent study design *(based on Zelen, N Engl J Med 1979)* (9)

The *WHISH* trial uses the randomized consent design proposed by Zelen (9) (Figure 1), in which eligible participants are randomized before being contacted and before informed consent is obtained. The design is based on the intention-to-treat principle; outcomes are assessed on the entire randomized population, regardless of their subsequent level of participation. This design is appropriate when the goal is to test the impact of a public health intervention on the population at large, not just within the subgroup of the population that is willing to participate, and tests the most critical research question: “Can a centrally delivered public health physical activity intervention reduce CVD events in a large population of older women?”

### Study Population and Eligibility Criteria

The *WHISH* trial is embedded in the WHI Extension Study (WHI-ES), which is continuing to collect health outcomes and related data on women who were originally enrolled in the WHI Clinical Trial Program or the Observational Study at 40 U.S. clinical sites from 1993 to 2005, and which is expected to continue through at least 2027. Women in the WHI-ES consented to continued participation and extended follow-up in 2004–2005 and again in 2010. Earlier phases of the WHI program have been described in detail previously (10–18).

Participants in the WHI-ES were eligible for *WHISH* if they were alive in 2015, and free of conditions that would limit their full participation, specifically known dementia, living in a nursing home, inability to walk, inability to read English, or unavailability of follow-up CVD outcomes data.

**Figure 2.**
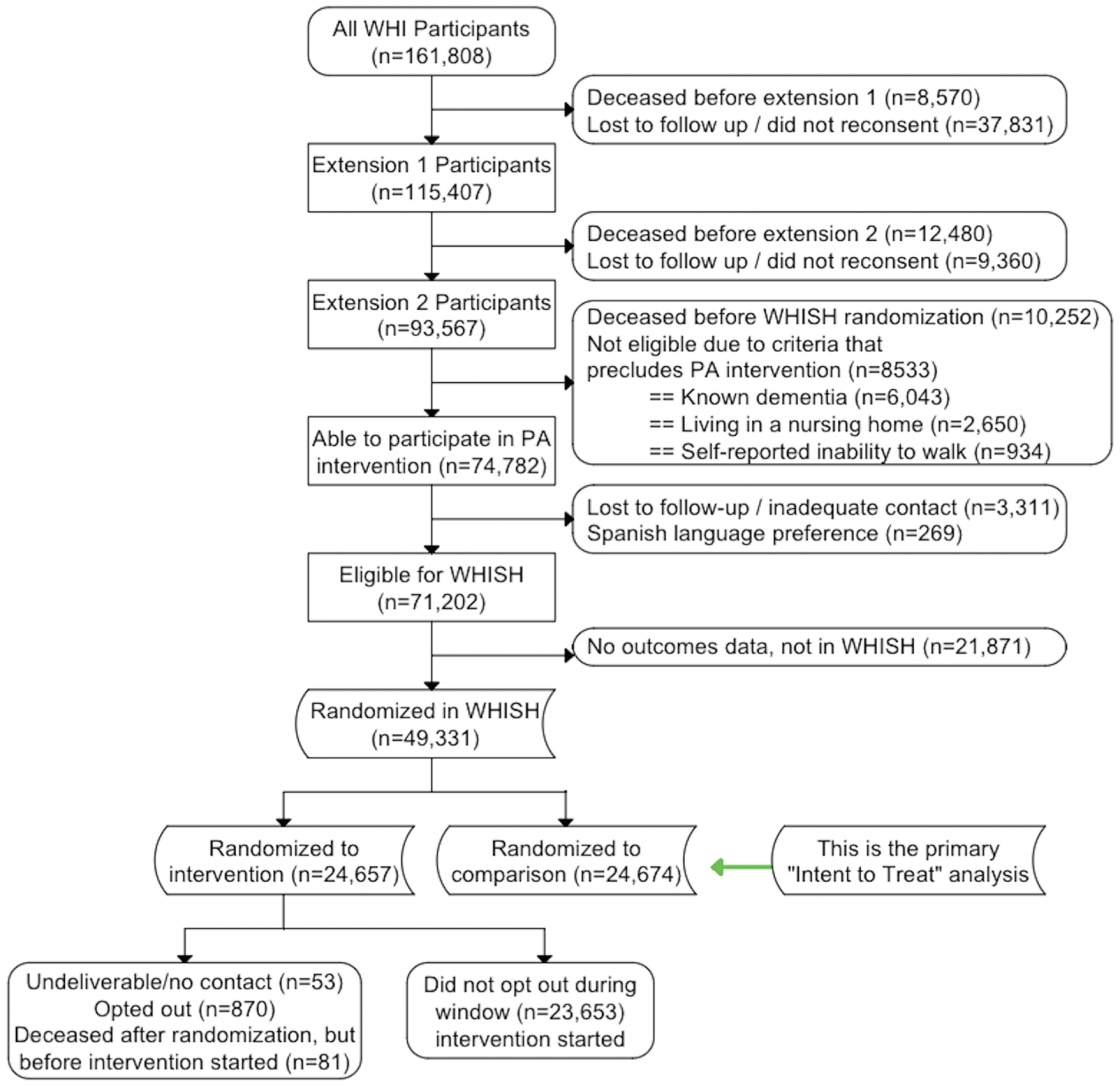
*WHISH* Trial Consort Diagram.

The *WHISH* Data Coordinating Center (DCC) used the WHI database to screen women for eligibility based on previously collected WHI data. Of the original 93 567 women who consented to ongoing follow-up in the WHI Extension Study in 2010, 10 252 were known to have died before the date of randomization into *WHISH*, and 8533 were excluded due to known dementia (*n* = 6043), living in a nursing home (*n* = 2650), self-reported inability to walk (*n* = 934), or Spanish language preference (*n* = 269). An additional 3311 participants had been lost to follow up due to inadequate contact information such as invalid addresses (Figure 2).

Availability of adjudicated CVD outcomes differed for 2 categories of WHI-ES participants. The “Medical Records Cohort (MRC)” is a subcohort in which CVD outcomes continued to be verified by review of medical records and physician-adjudication. The MRC cohort includes women who had participated in the WHI Hormone Trials, or were an African American or Hispanic participant in the Diet Modification Trial or Observational Study. All MRC cohort women were eligible for *WHISH* if they met other eligibility criteria as detailed above. The remaining WHI-ES women are part of the “Self-reported Outcomes Cohort (SRC)” and eligible for *WHISH* if they met the criteria above and their follow-up hospitalization data were available through linkage to the Centers for Medicare and Medicaid Services (CMS) database. Previous validation studies showed that CMS diagnosis codes had good agreement with WHI MRC adjudicated outcomes for MI (κ = 0.74) (19), stroke (κ = 0.84) (20), and other CVD outcomes (21,22). Unavailability of CMS follow-up for major CVD events excluded 21 871 SRC participants, leaving a total of 49 331 participants eligible for randomization (Figure 2).

The *WHISH* protocol is registered on clinical trials.gov (23) and was approved by Human Subjects Review committees at the Fred Hutchinson Cancer Research Center, Stanford University, and the University of California, San Diego.

### Randomization and Initial Contact

Before *WHISH* randomization, all WHI-ES participants were mailed a newsletter describing new studies underway for eligible WHI participants*. WHISH* was described as a study testing whether increasing physical activity would lower the risk of heart disease and help maintain independent living. Eligible WHI-ES participants were randomized using the Zelen design in equal numbers either to the *WHISH* physical activity intervention or to continue usual follow-up. For practical reasons, participants were randomized in 3 waves about 1 month apart. Randomization was carried out in 24 strata formed by baseline age in tertiles (<77, 77–82, and ≥83 years), 4 WHI regions, and the method of outcomes collection (MRC or CMS Medicare hospitalization data). Among WHI-ES participants who met other eligibility criteria, 16 251 were in the MRC and 30 346 had sufficient Medicare data to be included in *WHISH*. An additional 2734 SRC women had outcomes adjudicated by the WHI for other reasons, yielding a total of 49 331 women randomized into the *WHISH* Trial.

### Consent

Participants in the WHI-ES had all previously consented to follow up and collection of outcomes data. Women randomized in *WHISH* to usual follow-up received no further information about the trial. Women randomized to the physical activity intervention were sent an initial mailing from the *WHISH* DCC that included intervention materials, a *Go4Life* Workout to Go: Mini Exercise Guide (24), and a telephone number to call if they preferred to opt out of receiving additional physical activity materials. After removing 870 (3.5%) women who “opted out,” 53 who had undeliverable addresses and 81 who had died after randomization but before the intervention began, Stanford received names and contact information for 23 653 participants for the *WHISH* physical activity intervention.

Intervention participants received written information meeting HIPAA requirements in the fourth Stanford mailing (November 2015), and were provided a Stanford telephone number, postal address, and e-mail addresses to use if the participant did not consent to the use of data collected from surveys and postcards, data entered by participants into a *WHISH* website tracking tool, recorded through the *WHISH* automated telephone-based interactive voice response system, or returned as handwritten entries on annual *WHISH* calendars, or input received by telephone, e-mail, or other means. Intervention participants were reminded that they could request no further contact at any time in the future, thereby withdrawing passive consent, or request discontinuation of any specific component of the intervention, including but not limited to quarterly mailings, outbound monthly telephone messages, or emails.

### WHISH Physical Activity Intervention

The *WHISH* physical activity intervention goals are based on the U.S. Department of Health and Human Services 2008 Physical Activity Guidelines for older Americans (1), as presented in the National Institute on Aging (NIA) *Go4Life* health education campaign (25), and the updated 2018 Physical Activity Guidelines for older Americans (2). These federal initiatives emphasize increasing or maintaining aerobic physical activity (primarily walking) and decreasing sedentary behavior (especially sitting), as well as multicomponent physical activity recommendations regarding muscle-strengthening, balance, and flexibility. Based on state-of-the-science behavioral theories, including Social Cognitive Theory (26), the Transtheoretical Model of behavior change (27), and Stages of (Readiness of) Change (28), the intervention is delivered through multiple channels including quarterly (seasonal) *WHISH*ful Actions newsletters, with inserts targeted at 3 participant groups based on lower, middle and higher levels of self-reported physical functioning and physical activity levels; monthly outbound telephone calls with short (~1 minute) motivational messages; monthly motivational e-mails, for the approximately one-fourth of participants who provided email addresses; access to the *WHISH* website which includes videos of participants demonstrating exercises as well as many additional resources; and occasional personal contact with intervention staff by phone, e-mail, or regular mail as required or requested. The physical activity intervention adapts to participant input received from annual surveys or other inbound communication channels, regarding physical activity preferences, resources, barriers, and motivational drivers (29–31).

In the first year of the intervention, all physical activity participants received the *Go4Life Exercise & Physical Activity:* Your *Everyday Guide* (32), pedometers and calendars with instructions on how to track their physical activity, and resistance bands with examples of exercises using the bands for muscle-strengthening. In subsequent years, participants have received annual calendars, new pedometers and resistance bands, targeted inserts demonstrating various strength, balance, flexibility and endurance exercises, a *WHISH* sun visor, and chances to win gift cards by participating in special *WHISH* challenges, and they may request replacements of these materials as needed. Further specific details of the intervention program will be presented in a subsequent manuscript.

### Outcomes Ascertainment

The primary outcome of the *WHISH* trial is major CV events, defined as the first event since randomization in *WHISH* of either MI, stroke, or CV Death. The primary safety outcomes include non-CV death, hip fracture, other clinical (non-hip) fractures, and falls. Secondary outcomes are venous thromboembolism, peripheral artery disease, physical functioning as determined by the Rand-36 10-item score (33), and coronary revascularization (coronary artery bypass graft or percutaneous coronary intervention) as the secondary safety outcome. Additional outcomes, including cancer, heart failure, and a wide array of clinical diagnoses, are ascertained on all participants through CMS linkage and annual WHI surveillance (34). Two ancillary studies to the *WHISH* Trial have been funded specifically to adjudicate heart failure and atrial fibrillation endpoints (R01HL130591, CB Eaton, Principal Investigator, and R01HL136390, MV Perez, Principal Investigator).

Details of the WHI outcomes ascertainment and adjudication procedures have been published (34,35) with modifications presented in the WHI-ES Protocol (36). Briefly, WHI-ES participants are followed annually, primarily by mailed questionnaires, to collect data on self-reported health events and related conditions. Mailings include a *Form 33 - Medical History Update* (Supplementary Appendix A) and additional questionnaires (see below), which most participants (or their proxies) return by mail. WHI staff may collect Form 33 by phone for women who have not returned it after repeat mailings.

Outcomes ascertainment procedures for *WHISH* depend on the outcome and whether the participant is in the MRC or in Medicare. These procedures use a combination of self-report and adjudicated events, as well as National Death Index and CMS linkages to identify outcome events (Table 1).

**Table 1.** Methods Used to Ascertain Outcomes in the *WHISH* Trial

	MRC Participants^a^	Participants With Medicare Part A+B or A Only^b^
Numbers at time of randomization	*N* = 18 985	*N* = 30 346
Outcome	Outcomes ascertainment procedures	
Coronary heart disease, revascularization, heart valve problem/repair, aortic aneurysm, stroke, carotid artery disease, heart failure, atrial fibrillation, venous thromboembolism, peripheral artery disease, hip fracture	Full clinical outcomes ascertainment and documentation, including physician-adjudicated medical records review (full review)	From CMS codes using established algorithms
Death	National Death Index, medical records, and adjudication	National Death Index
Cancer (all sites except nonmelanoma skin cancer),	Full review	Full review
Angina, transient ischemic attack, other fractures and several other age-related diseases, including chronic obstructive pulmonary disease, diabetes mellitus requiring therapy, hypertension requiring therapy, intestinal or colon polyps or adenomas, macular degeneration, osteoarthritis or arthritis with aging, Parkinson’s disease, systemic lupus erythematosus, moderate/severe memory problems (dementia/Alzheimer’s), falls, hysterectomy	Self-report	Self-report
Physical functioning (10-item Rand-36 scale)	Self-report	Self-report

*Note*: CMS = Centers for Medicare and Medicaid Services; MRC = Medical records cohort.

^a^This group includes participants with Centers for Medicare and Medicaid Services (CMS) data at time of the start of the trial who have changed insurance status. ^b^This group includes participants with Medicare Part A+B or A only at the time of the last release of Medicare data by CMS before the start of the trial, and WHI Medical Records Cohort (MRC) participants with Medicare who do not return forms.

An overlap of 8431 participants in the MRC with CMS linkage allows a comparison of outcomes from the 2 sources. Highly concordant results reported from these sources in WHI for MI (19), stroke (20), peripheral vascular events (22), and venous thromboembolism (21) suggest that it is possible to combine adjudicated and CMS data to monitor outcomes in the *WHISH* trial.

### Physical Functioning Assessment

Physical functioning (PF) is based on participant responses to questions in an annual WHI form (*Form 151 Activities of Daily Living*, Supplementary Appendix B), which is mailed with *Form 33,* that ask about limitations (no, not limited at all; yes, limited a little; yes, limited a lot) regarding 10 specific activities (Q 7–16) from RAND-36 (33). The PF score ranges from 0 to 100 (in 5-point increments), with 100 meaning no limitations on any activities.

### Physical Activity Assessment

Physical Activity assessment is based on both a self-reported physical activity questionnaire (*Form 521 Physical Activity Questionnaire*, Supplementary Appendix C), which is mailed annually to all *WHISH* participants with *Form 33* and labeled as a WHI form, to maintain blinding and reduce response bias, and through periodic accelerometry in a subsample of *WHISH* participants (see below). *Form 521* ascertains walking, sedentary time, sleeping, details about falls, and an abbreviated CHAMPS (37,38) questionnaire. Whereas *WHISH* investigators are blinded regarding clinical outcomes, with the exception of Dr.Kooperberg, (see Monitoring), trial investigators regularly review the comparisons of self-reported physical activity data as a process measure for the intervention materials and adapt the intervention as appropriate.

A previous WHI ancillary study, the Objective Physical Activity and Cardiovascular Health (OPACH) Study (R01 HL105065, AZ LaCroix, PI) provided an opportunity to conduct a substudy of accelerometry within a subset of 4730 *WHISH* participants who had successfully worn and returned Actigraph GT3X+ accelerometers in 2012–2014, thereby providing pre-*WHISH* Trial data for comparison between randomized groups (39). Surviving *WHISH*-OPACH participants who had returned usable accelerometer data were invited to join and actively consent to participation in the “WHI Physical Activity Follow-up Study” with no reference to the *WHISH* trial. To achieve balanced groups of at least 1000 women in both the *WHISH* arms throughout the planned follow-up period, the recruitment target was set at 2300; 2349 women consented. Of these, 85.4% (*n* = 2007) returned accelerometers in Year 1 of *WHISH*. Among those who were still alive, 75.1% (*n* = 1731) returned accelerometers in Year 2, and 63.7% *n* = 1394) returned them at Year 4. Details of Accelerometer Substudy are provided in Supplementary Appendix D.

### Trial Monitoring

An NHLBI-appointed Data Safety and Monitoring Board (DSMB) meets annually and receives updates on the physical activity intervention, including measurements of participant engagement, and monitors self-reported and accelerometer-measured physical activity and clinical outcomes data.

In early discussions, the DSMB determined that the trial would not have formal monitoring boundaries for stopping early for futility with respect to the CVD outcomes in recognition of the potential value of increasing or maintaining physical activity on multiple health conditions. The DSMB also suggested that the trial not have monitoring boundaries for stopping early for CVD efficacy because the benefit of getting a definitive answer will likely outweigh any benefit of making a less clear result public. This decision was justified based on several considerations: (i) the need for a broader assessment of the benefits and risks of increasing physical activity in older women; (ii) a strong anticipation of CVD benefits from prior studies of physical activity (4,5); and (iii) the availability to the general public including the *WHISH* usual follow-up comparison group of the NIA *Go4Life* materials (24,25,32) which were provided to the physical activity intervention group. Thus, trial monitoring for *WHISH* focuses on 2 main purposes: assuring the safety of participants; and testing the primary and secondary hypotheses adequately. *WHISH* could be stopped if the intervention group does not appear to be more physically active or less sedentary than the comparison group, which could result in a noninformative test. In addition, the NHLBI has requested an interim monitoring for benefit and futility in Spring 2022.

The primary outcome of CVD is also monitored as a (severe) safety outcome and together with non-CV death and hip fracture is evaluated annually, that is, each of these 3 outcomes are monitored at each annual interim meeting using a Z-value of 2.42. Based on simulations, the cumulative probability that the Z-statistic for one of these outcomes will exceed 2.42 at any meeting, if in truth there is no effect, is approximately 5%, assuming that these 3 outcomes are independent. The primary safety aims, total clinical fracture, hip fracture, and falls are also monitored, whereas less severe outcomes, including coronary revascularization, falls, and other clinical fractures, are provided regularly but not formally monitored because the clinical implications for these outcomes may or may not be apparent at the time they are observed. For example, increased physical activity in the intervention group may uncover symptoms of angina or claudication, which could lead to revascularization procedures which are intended to reduce the rate of subsequent, more serious events such as MI, and hence do not carry the same adverse safety signal as other events being monitored.

### Duration of Follow-up and Statistical Considerations

**Figure 3.**
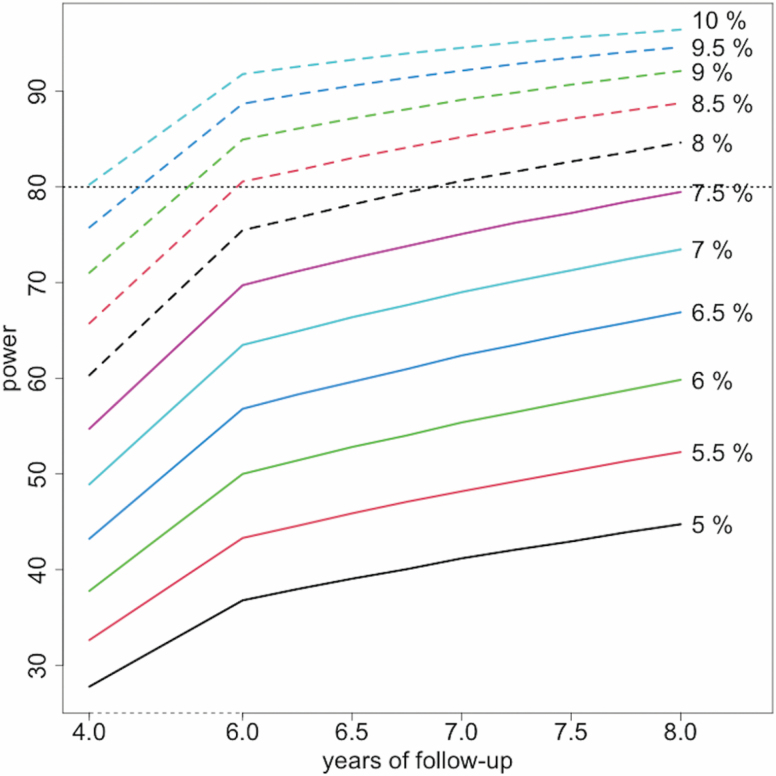
Estimated power for the *WHISH* primary endpoint as a function of the observed effect size of physical activity.

The *WHISH* trial was funded for an initial 5 years (2015–2020). Renewal funding will extend the follow-up to approximately 9 years (to September 2024). Power calculations for the renewal of the *WHISH* trial were based on the observed effects of the *WHISH* physical activity intervention, the observed event rates for the intervention and comparison arms combined, and the observed relation between physical activity and CVD from the OPACH study. The OPACH study was also carried out in WHI participants and should, thus, give us the best possible effect estimates. As of February 1, 2020, *WHISH* had 4 years of follow-up, the renewal would extend that follow-up to 8 years. The current difference in MET-hours/week walking, as reported by the complete cohort, is over 8.5%, and has been increasing during the study. The currently observed event rates as a function of age for the primary *WHISH* outcome (CHD, stroke, CVD death), and the “censoring outcome” (death from other causes) is estimated from the observed data, combining both arms. The observational OPACH study obtained a hazard ratio of 0.79 for the *WHISH* CVD outcome, for a 1-hour increment of light physical activity which is comparable to nonpurposeful (daily life) walking in the age range of women studied in *WHISH*. In OPACH, the average light physical activity was 4.76 hours/day, so an 8.5% intervention group difference is equivalent to 0.4 hours/day, corresponding to a 9% decreased rate of the primary CVD outcome. Using the age distribution of *WHISH* participants and the observed event and censoring rates, we obtain the power estimates shown in Figure 3. We note that with an intervention effect of 9% we have over 90% power, and with an effect of 8% we have close to 85% power after 8 years.

For each of the primary, safety, and secondary outcomes, intervention effect sizes, confidence intervals, and Z-statistics are based on Cox-proportional hazards models, with the exceptions of falls, which are compared using Poisson regression, and physical function, for which the mean difference between the groups is compared using linear regression. All analyses are stratified by categories applied to the randomization of participants, including age on April 1, 2015 (in tertiles), WHI region, and outcomes data source (MRC vs CMS fee-for-service). Using standard risk sets in survival analysis, participants who change from fee-for-service to managed care will have their self-reports adjudicated after the switch and are moved to the MRC stratum at the time of the switch in insurance coverage. Similarly, outcomes for participants who have 2 types of data, but do not return their self-report Form 33, will be assessed using CMS data after their last returned Form 33.

All analyses will be carried out using an intention-to-treat analysis. Participants randomized to the intervention who “opted out” or requested that the intervention be stopped as the trial progressed will be included in all analyses, as assigned, for monitoring and primary reporting purposes. Deaths that occurred between randomization and the start of intervention are also included in the analyses.

## Results

### Baseline Characteristics of Randomized Participants

*WHISH* Trial Baseline characteristics for women assigned to the Intervention (*N* = 24 657) versus Control (*N* = 24 674) were well balanced by randomized intervention group (Table 2). The average age of women was 80 years with 9% Black or African American (*n* = 4514), 3.3% Hispanic/Latina (*n* = 1628), 2% Asian (*n* = 923), and 84% non-Hispanic White. Average body mass index based on the most recent WHI clinic visit (no later than 2005) was 28 kg/m^2^, with 30% of all *WHISH* participants in the obese range, ≥30 kg/m^2^. Use of blood pressure medications at WHI baseline was common (61%), 44% were taking lipid medication, 17% self-reported a history of having diabetes requiring treatment, and 10.2% had history of CVD. At *WHISH* baseline (2015), about 20% of women reported being limited in walking one block a little or a lot and 10% had a previous history of CVD defined as MI, coronary artery bypass graft/percutaneous coronary intervention, or stroke. Baseline characteristics varied by baseline age tertiles for most variables including race/ethnic distribution (Table 3).

**Table 2.** Descriptive Statistics of *WHISH* Participants—by Trial Arm

Category	All (*n* = 49 331)		Intervention (*n* = 24 657)		Control (*n* = 24 674)		*p* Value
	*n*	%	*n*	%	*n*	%	
Outcome Coverage							.76
WHI-adjudicated only	10 554	21.4	5306	21.5	5248	21.3	
CMS only	30 346	61.5	15 156	61.5	15 190	61.6	
WHI + CMS	8431	17.1	4195	17.0	4236	17.2	
Age, mean (*SD*)	79.7	(6.2)	79.8	(6.2)	79.7	(6.2)	.45
<77	16 878	34.2	8421	34.2	8457	34.3	
77–82	16 220	32.9	8122	32.9	8098	32.8	
≥83	16 233	32.9	8114	32.9	8119	32.9	
Ethnicity							.93
Non-Hispanic White	41 606	84.3	20 771	84.2	20 835	84.4	
Non-Hispanic Black/African American	4514	9.2	2276	9.2	2238	9.1	
Hispanic/Latina	1628	3.3	819	3.3	809	3.3	
American Indian/Alaskan Native	151	0.3	71	0.3	80	0.3	
Asian/Pacific Islander	923	1.9	469	1.9	454	1.8	
Unknown	509	1.0	251	1.0	258	1.0	
Current smoker							>.99
Yes	1216	2.5	607	2.5	609	2.5	
No	48 103	97.5	24 044	97.5	24 059	97.5	
Use of BP Meds							.41
Yes	30 136	61.1	15 018	60.9	15 118	61.3	
No	19 195	38.9	9639	39.1	9556	38.7	
Use of Lipids Meds							.64
Yes	21 801	44.2	10 871	44.1	10 930	44.3	
No	27 530	55.8	13 786	55.9	13 744	55.7	
BMI, mean (*SD*)^a^	28.0	(5.8)	28.0	(5.8)	28.0	(5.8)	.67
>30	14 921	30.2	7432	30.1	7489	30.4	
≤30	34 383	69.7	17 210	69.8	17 173	69.6	
Physical Functioning Score, mean (*SD*)	71.6	(25.2)	71.5	(25.2)	71.6	(25.2)	.76
<65	15 152	30.7	7601	30.8	7551	30.6	
65–75	8283	16.8	4119	16.7	4164	16.9	
76–89	8312	16.8	4163	16.9	4149	16.8	
≥90	17 525	35.5	8742	35.5	8783	35.6	
Limited ability to go up one flight of stairs							.83
No, not limited	35 051	71.1	17 539	71.1	17 512	71.0	
Yes, limited a little	10 777	21.8	5378	21.8	5399	21.9	
Yes, limited a lot	3497	7.1	1736	7.0	1761	7.1	
Limited ability to walk one block							.58
No, not limited	38 899	78.9	19 380	78.6	19 519	79.1	
Yes, limited a little	7294	14.8	3714	15.1	3580	14.5	
Yes, limited a lot	3130	6.3	1559	6.3	1571	6.4	
Episodes of exercise, mean (*SD*)	4.7	(3.9)	4.7	(3.9)	4.7	(3.9)	.86
0–2	15 137	30.7	7527	30.5	7610	30.8	
>2–6	18 920	38.4	9511	38.6	9409	38.1	
>6	15 274	31.0	7619	30.9	7655	31.0	
Self-report treated diabetes ever	8446	17.1	4205	17.1	4241	17.2	.69
WHI outcomes							
CVD	5041	10.2	2542	10.3	2499	10.1	.51
MI	1799	3.6	927	3.8	872	3.5	.18
CABG/PCI	3030	6.1	1537	6.2	1493	6.1	.40
Stroke	1632	3.3	811	3.3	821	3.3	.81
WHI HT Trial Arm							.88
Active	5736	11.6	2868	11.6	2868	11.6	
Placebo	5645	11.4	2804	11.4	2841	11.5	
Not randomized	37 950	76.9	18 985	77.0	18 965	76.9	
WHI DM Trial Arm							.66
Intervention	6402	13.0	3203	13.0	3199	13.0	
Comparison	9932	20.1	5003	20.3	4929	20.0	
Not randomized	32 997	66.9	16 451	66.7	16 546	67.1	
WHI CaD Trial Arm							.09
Active	7369	14.9	3656	14.8	3713	15.0	
Placebo	7079	14.4	3623	14.7	3456	14.0	
Not randomized	34 883	70.7	17 378	70.5	17 505	70.9	
WHI study component							.35
Clinical Trial	24 186	49.0	12 141	49.2	12045	48.8	
Observational study	25 145	51.0	12 516	50.8	12 629	51.2	

*Note*: BMI = Body mass index; BP = Blood pressure; CABG = Coronary artery bypass graft; CaD = Calcium/Vitamin D Supplementation; CMS = Centers for Medicare and Medicaid Services; CVD = Cardiovascular disease; DM = Dietary modification; HT = Hormone Trial; MRC = Medical records cohort; PCI = Percutaneous coronary intervention; SD = Standard deviation.

^a^BMI is based on the most recent WHI clinic visit weight and height, none of which were after 2005.

**Table 3.** Descriptive Statistics of *WHISH* Participants—by Baseline Age Tertiles

Category	<77 (*n* = 16 878)		77–82 (*n* = 16 220)		≥83 (*n* = 16 233)	
	*n*	%	*n*	%	*n*	%
Outcome coverage						
WHI-adjudicated only	3849	22.8	3493	21.5	3212	19.8
CMS only	10 094	59.8	9980	61.5	10 272	63.3
WHI + CMS	2935	17.4	2747	16.9	2749	16.9
Age, mean (*SD*)	73.1	(2.2)	79.4	(1.7)	87.0	(3.2)
Ethnicity						
Non-Hispanic White	13 544	80.2	13 735	84.7	14 327	88.3
Non-Hispanic Black/African American	1946	11.5	1493	9.2	1075	6.6
Hispanic/Latina	771	4.6	502	3.1	355	2.2
American Indian/Alaskan Native	73	0.4	45	0.3	33	0.2
Asian/Pacific Islander	375	2.2	277	1.7	271	1.7
Unknown	169	1.0	168	1.0	172	1.1
Current smoker						
Yes	603	3.6	385	2.4	228	1.4
No	16 268	96.4	15 831	97.6	16 004	98.6
Use of BP Meds						
Yes	8770	52.0	9989	61.6	11 377	70.1
No	8108	48.0	6231	38.4	4856	29.9
Use of Lipids Meds						
Yes	6841	40.5	7464	46.0	7496	46.2
No	10 037	59.5	8756	54.0	8737	53.8
BMI, mean (*SD*)^a^	28.4	(6.3)	28.2	(5.8)	27.4	(5.2)
>30	5561	32.9	5077	31.3	4283	26.4
≤30	11 305	67.0	11 135	68.6	11 943	73.6
Physical Functioning Score, mean (*SD*)	79.3	(21.8)	72.3	(24.1)	62.7	(26.6)
<65	3241	19.2	4720	29.1	7191	44.3
65–75	2382	14.1	2894	17.8	3007	18.5
76–89	2921	17.3	2929	18.1	2462	15.2
≥90	8328	49.3	5657	34.9	3540	21.8
Limited ability to go up one flight of stairs						
No, not limited	13 609	80.6	11 762	72.5	9680	59.6
Yes, limited a little	2650	15.7	3507	21.6	4620	28.5
Yes, limited a lot	619	3.7	948	5.8	1930	11.9
Limited ability to walk one block						
No, not limited	14 854	88.0	12 991	80.1	11 054	68.1
Yes, limited a little	1482	8.8	2330	14.4	3482	21.5
Yes, limited a lot	542	3.2	897	5.5	1691	10.4
Episodes of exercise, mean (*SD*)	5.2	(4.0)	4.7	(3.9)	4.1	(3.8)
0–2	4381	26.0	4809	29.6	5947	36.6
>2–6	6419	38.0	6331	39.0	6170	38.0
>6	6078	36.0	5080	31.3	4116	25.4
Self-report treated diabetes ever	2922	17.3	2906	17.9	2618	16.1
WHI outcomes						
CVD	1005	6.0	1638	10.1	2398	14.8
MI	341	2.0	578	3.6	880	5.4
CABG/PCI	620	3.7	1001	6.2	1409	8.7
Stroke	301	1.8	518	3.2	813	5.0
WHI HT Trial Arm						
Active	1841	10.9	1881	11.6	2014	12.4
Placebo	1743	10.3	1863	11.5	2039	12.6
Not randomized	13 294	78.8	12 476	76.9	12 180	75.0
WHI DM Trial Arm						
Intervention	2221	13.2	2252	13.9	1929	11.9
Comparison	3507	20.8	3508	21.6	2917	18.0
Not randomized	11 150	66.1	10 460	64.5	11 387	70.1
WHI CaD Trial Arm						
Active	2572	15.2	2514	15.5	2283	14.1
Placebo	2439	14.5	2436	15.0	2204	13.6
Not randomized	11 867	70.3	11 270	69.5	11 746	72.4
WHI study component						
Clinical trial	8096	48.0	8297	51.2	7793	48.0
Observational study	8782	52.0	7923	48.8	8440	52.0

*Note*: BMI = Body mass index; BP = Blood pressure; CABG = Coronary artery bypass graft; CaD = Calcium/Vitamin D Supplementation; CMS = Centers for Medicare and Medicaid Services; CVD = Cardiovascular disease; DM = Dietary modification; HT = Hormone trial; MRC = Medical records cohort; PCI = Percutaneous coronary intervention; SD = Standard deviation.

^a^BMI is based on the most recent WHI clinic visit weight and height, none of which were after 2005.

## Discussion

The *WHISH* trial is the first randomized, controlled trial to test whether an intervention designed specifically to increase physical activity and reduce sedentary behavior, independent of diet, weight loss, or other interventions, will reduce major CVD outcomes. As it is specifically testing this hypothesis in older women, additional aims focus on determining whether the intervention benefits physical function and other issues of particular importance to older adults, including maintaining independence and mobility, and its safety with respect to non-CVD mortality and falls and fractures. By recognizing the opportunity to utilize a randomized consent design (9) in the large multiethnic and well-characterized WHI cohort of older women, the *WHISH* trial meets the challenge posed by NHLBI leadership to design highly efficient pragmatic clinical trials, embedded within ongoing epidemiologic cohort studies, to test hypotheses of major public health significance (40,41). Utilization of materials available through the National Institute of Aging *Go4Life* program and website (24,25,32) to deliver an intervention based on accepted physical activity recommendations for all older Americans (1,2,42) adds to the efficiency and potential scalability of the *WHISH* trial program being tested.

The consistent evidence to date that physical activity reduces CVD mortality and incidence, including coronary heart disease and stroke, is based on a large body of observational data studying these endpoints, as well as randomized controlled trials focusing on CV risk factors rather than clinical outcomes (4,5). It is, of course, recognized that physical activity has many health benefits, with sufficient rigorous evidence irrespective of CVD reduction to recommend physical activity as part of a broader public health framework (1,2). However, because CVD deaths in women aged 80 years and older account for two thirds of all CVD deaths in U.S. women, experimental evidence that physical activity reduces CVD in this population would have extraordinary public health significance.

The randomized consent design (9) in the *WHISH* trial removes a serious bias of the majority of previous physical activity trials which attract and recruit individuals whose willingness to increase activity may be directly related to an inherent physiology that yields greater benefit than what the general population is likely to experience. Many such trials have excluded individuals who are already active but might benefit from even further physical activity change or reduction in time spent sedentary. While some portion of WHI-ES participants were initially attracted to the WHI hormone therapy or diet modification trials, and were likely more health-oriented than the general population of women their ages, use of the randomized consent design basically ensures that none of the *WHISH* participants joined because of their physical activity habits. Another novel design element that increased the efficiency of this pragmatic trial was the “opt out,” passive consent process which resulted in >96% of women randomly assigned to the intervention receiving the intervention.

The *WHISH* Trial accelerometry substudy took advantage of the fact that a large number of *WHISH* participants had participated in the OPACH study and had a first accelerometer wear in 2012–2014 before WHISH randomization. This is another example of a highly efficient study design leading to a truly rare sequence of 4 longitudinal accelerometer measures to evaluate trajectories of physical activity over time by *WHISH* randomization group.

In conclusion, the pragmatic *WHISH* trial is an innovative physical activity trial with the potential to provide strong evidence of physical activity as a sole modality to reduce CVD in older adults. If this easily scalable intervention proves to be safe and effective in improving cardiovascular health and other outcomes of importance to older women, the *WHISH* physical activity intervention can be widely disseminated, as well as adapted to meet the needs of many additional segments of the U.S. population.

## Supplementary Material

glaa325_suppl_Supplementary_Appendix_A

glaa325_suppl_Supplementary_Appendix_B

glaa325_suppl_Supplementary_Appendix_C

glaa325_suppl_Supplementary_Appendix_D
