# Supplementary material for: Women’s Health Initiative Strong and Healthy Pragmatic Physical Activity Intervention Trial for Cardiovascular Disease Prevention: Design and Baseline Characteristics
Source: J Gerontol A Biol Sci Med Sci. 2021 Jan 12;76(4):725–34. doi: 10.1093/gerona/glaa325 (PMC8011700; doi:10.1093/gerona/glaa325)
Supplement: glaa325_suppl_Supplementary_Appendix_A [file glaa325_suppl_supplementary_appendix_a.pdf]

Form 33 - Medical History Update

Please use a pencil or black pen only to complete this form.

This form asks about any health problems and health care since:

____/____/________
MM DD YYYY

Do not report events that happened before the date above. However, if you are not sure of a date and don't think that you have reported it to us before, please answer the questions.

1. **What is today's date?** Write date here: __ __/__ __/__ __
Month Day Year

Mark the month, day and year below. **Mark only one bubble per line.**

Month ○ ○ ○ ○ ○ ○ ○ ○ ○ ○ ○ ○
1 2 3 4 5 6 7 8 9 10 11 12

Day ○
1 2 3 4 5 6 7 8 9 10 11 12 13 14 15 16 17 18 19 20 21 22 23 24 25 26 27 28 29 30 31

Year ○ ○ ○ ○ ○ ○
20 21 22 23 24 25

2. **Who is completing this form?**
   - ○ 1 Self (WHI Study participant)
   - ○ 2 Other, on behalf of the WHI participant
     Name and relationship to participant: ________________________________

3. Best phone number to reach the person completing this form: (_____) ______ _______

OFFICE USE ONLY

1. Date Received:
____/____/________
MM DD YYYY

2. Reviewed By: 80 - __ __ __

○ RCR ○ OU1 ○ OU2

3. Contact Type:
○ 1 Phone
○ 2 Mail
○ 8 Other

4. Visit Type:
○ 3 Annual
○ 4 Non-Routine

AFFIX LABEL BETWEEN LINES

Participant ID Label

PLEASE MAKE NO MARKS IN THIS AREA

4. Since the date on the front of this form, have you had any of the following exams, tests, or procedures done by a doctor or other health care provider? **Mark all that apply.**

- ○ 1 Breast exam
- ○ 2 Mammogram
- ○ 3 Test of breast tissue or fluid for disease (breast biopsy or aspiration)
- ○ 4 Other breast examination tests such as MRI or ultrasound
- ○ 5 Test for the presence of blood in your stool or bowel movement (Hemoccult®, guaiac, Cologuard®)
- ○ 6 Tube inserted into your bowel to check for bowel problems (sigmoidoscopy or colonoscopy)
- ○ 7 Hysterectomy (surgery to remove the uterus or womb)
- ○ 8 Biopsy of the endometrium (lining of the uterus or womb)
- ○ 99 **None of the above apply**

5. Since the date on the front of this form, has a doctor or other health care provider told you that you have any of the following conditions? **Mark all that apply.**

- ○ 1 Chronic obstructive pulmonary disease (COPD), emphysema, or chronic bronchitis
- ○ 2 Angina or chest pain from a heart condition for which you were hospitalized for one night or more (not a heart attack)
- ○ 3 Transient ischemic attack (not a stroke)
- ○ 4 Osteoarthritis or arthritis associated with aging
- ○ 5 Macular degeneration associated with aging
- ○ 6 Moderate or severe memory problems
- ○ 7 Dementia or Alzheimer's
- ○ 8 Parkinson's disease
- ○ 9 Intestine or colon polyps or adenomas
- ○ 99 **None of the above apply**

6. Since the date on the front of this form, has a doctor or other health care provider prescribed any of the following treatments for diabetes? **Mark all that apply.**

- ○ 1 Insulin
- ○ 2 Pills or medications other than insulin
- ○ 3 Diet and/or physical activity
- ○ 99 **None of the above apply** (I do not have or no longer have diabetes.)

7. Since the date on the front of this form, has a doctor or other health care provider prescribed for the first time pills for high blood pressure or hypertension?

○ 1 Yes ○ 0 No

8. Since the date on the front of this form, how many times did you fall and land on the floor or ground? Do not include falls due to sports. **Mark only one.**

○ 0 None ○ 1 One time ○ 2 Two times ○ 3 Three or more times

8.1 Were you injured as a result of any falls? ○ 1 Yes ○ 0 No

Since the date on the front of this form, have you been diagnosed or treated for any of the following conditions or procedures? **Mark Yes or No for each item.**

	Yes	No
**9.** Stroke	○ 1	○ 0
**10.** MI, heart attack (coronary, myocardial infarction)	○ 1	○ 0
**11.** Heart failure (congestive heart failure, CHF or HF)	○ 1	○ 0
**12.** Heart bypass operation (coronary bypass surgery or CABG)	○ 1	○ 0
**13.** Heart valve problem or surgery to repair or replace a heart valve	○ 1	○ 0
**14.** Abdominal aortic aneurysm (AAA) requiring surgery or stent	○ 1	○ 0
**15.** Procedure or surgery to unblock narrowed blood vessels in your neck (carotid endarterectomy, carotid angioplasty, or carotid stent)	○ 1	○ 0
**16.** Poor blood circulation or any procedure to unblock narrowed arteries to your legs or feet (claudication, peripheral arterial disease, PAD, or gangrene). Do not include varicose veins.	○ 1	○ 0
**17.** Blood clots in your lungs (pulmonary embolism or PE)	○ 1	○ 0
**18.** Blood clots in the veins of your legs (deep vein thrombosis or DVT)	○ 1	○ 0
**19.** Atrial fibrillation, atrial flutter, or irregular heartbeat, requiring medications OR a procedure (such as electrical shock, cardioversion, ablation, or surgery)	○ 1	○ 0
**20.** Procedure to unblock narrowed blood vessels to your heart (opening the arteries of the heart with a stent, balloon, laser, or other device). Also called PTCA, angioplasty, or percutaneous coronary intervention (PCI).	○ 1	○ 0
**21.** Other heart or circulation conditions. Specify:________________________	○ 1	○ 0

PLEASE DO NOT WRITE IN THIS AREA

If you marked Yes to any of the heart or circulation items in questions 9-21, complete the health care provider information below. If not, go to Question 28 on the next page.

22. 1st hospital or doctor's office where you were diagnosed, treated, or admitted.

Facility name: ____________________

____
Street City State

23. Date you were diagnosed, treated, or admitted to a hospital: __ __-__ __-__ __
(Estimate if unsure.) Month Day Year

23.1 For what condition: ____________________

24. Were you hospitalized? ○ 1 Yes ○ 0 No → **Go to Question 25.**

24.1 How many nights? __ __ Nights (write "0" if no nights)

25. 2nd hospital or doctor's office where you were diagnosed, treated, or admitted.

Facility name: ____________________

____
Street City State

26. Date you were diagnosed, treated, or admitted to a hospital: __ __-__ __-__ __
(Estimate if unsure.) Month Day Year

26.1 For what condition: ____________________

27. Were you hospitalized? ○ 1 Yes ○ 0 No → **Go to Question 28 on next page.**

27.1 How many nights? __ __ Nights (write "0" if no nights)

Record any additional provider information in the Comments section at the end of this form, then continue to the next page.

28. Since the date on the front of this form, has a doctor or other health care provider told you that you have a new cancer, malignant growth, or tumor? Do not include benign tumors.

- ○1 Yes
- ○0 No → **Go to Question 29 on page 7.**

28.1 What type of new cancer? **Mark all that apply.**

- ○1 Breast
- ○2 Ovary
- ○3 Endometrium (lining of the uterus or womb)
- ○4 Cervix
- ○5 Other female genital organs (not ovary, endometrium, or cervix)
- ○6 Colon or rectum
- ○7 Bladder or urinary tract
- ○8 Brain
- ○9 Esophagus
- ○10 Gallbladder or bile ducts
- ○11 Hodgkin's lymphoma
- ○12 Non-Hodgkin's lymphoma
- ○13 Kidney
- ○14 Leukemia
- ○15 Liver
- ○16 Lung
- ○17 Melanoma
- ○18 Multiple myeloma
- ○19 Pancreas
- ○20 Skin cancer (not melanoma)
- ○21 Stomach
- ○22 Thyroid
- ○88 Other or unknown cancer
  Specify: ____________________
  ____
  ____

Complete the diagnosis information for the first new cancer.

28.2 When was this cancer diagnosed (estimate if unsure)? __ __-__ __-__ __
Month Day Year

28.3 Who was the doctor or other health care provider who diagnosed this cancer and at what facility was this cancer first diagnosed?

Doctor or provider name: ____________________

Facility name: ____________________

____
Street City State

28.4 What is the name of your oncologist? ____________________

28.5 Was an outpatient X-ray or imaging scan (CT, MRI, mammogram, bone or PET scan) taken to diagnose the cancer?

○ 1 Yes ○ 0 No → **Go to Question 28.8.**

28.6 Facility name: ______________________________

____
Street City State

28.7 Date of X-ray or scan (estimate if unsure): __ __-__ __-__ __
Month Day Year

Cancer-related surgeries for the first new cancer.

28.8 Since the date on the front of this form, have you had any cancer-related surgeries following the diagnosis of the first cancer?

○ 1 Yes ○ 0 No → **28.9** If No, are any planned?

○ 1 Yes
○ 0 No
→ **Go to Question 29 on the next page.**

Since the date on the front of this form:

28.10 Number of cancer-related surgeries you had: __ __

28.11 At what facility was this first cancer-related surgery done?

Facility name: ______________________________

____
Street City State

28.12 Date of first cancer-related surgery (estimate if unsure): __ __-__ __-__ __
Month Day Year

Record any additional provider information in the Comments section at the end of this form, then continue to the next page.

PLEASE DO NOT WRITE IN THIS AREA

29. Since the date on the front of this form, has a doctor or other health care provider told you that you have a new broken, fractured, or crushed hip or upper leg bone?

○ 1 Yes ○ 0 No ⟶ **Go to Question 30 on the next page.**

29.1 Which bone(s) did you break, fracture, or crush? **Mark all that apply.**

○ 1 Hip

○ 2 Upper leg (not hip)

29.2 Was this broken, fractured, or crushed hip or upper leg bone first diagnosed or treated during a hospital stay?

○ 1 Yes ○ 0 No ⟶ **Go to Question 29.6.**

29.3 In what hospital or medical facility were you diagnosed or treated for the broken, fractured, or crushed hip or upper leg bone?

Facility name: ____________________

____

Street City State

29.4 Date you entered the hospital (estimate if unsure): __ __-__ __-__ __

Month Day Year

29.5 Did you stay overnight? ○ 1 Yes ○ 0 No

29.6 **Was an outpatient X-ray or imaging scan** (CT or MRI) taken to diagnose the broken, fractured, or crushed hip or upper leg bone at a facility not reported above?

○ 1 Yes ○ 0 No ⟶ **Go to Question 30 on the next page.**

29.7 In what hospital or medical facility were you diagnosed or treated for the broken, fractured, or crushed hip or upper leg bone?

Facility name: ____________________

____

Street City State

29.8 Date of X-ray or other imaging scan (CT or MRI) (estimate if unsure): __ __-__ __-__ __

Month Day Year

30. Since the date on the front of this form, has a doctor or other health care provider told you that you have a new broken, fractured, or crushed bone **other than** hip or upper leg?

○ 1 Yes ○ 0 No

30.1 Which bone(s) did you break, fracture, or crush? **Mark all that apply.**

○ 1 Pelvis
○ 2 Knee (patella)
○ 3 Lower leg or ankle
○ 4 Foot (not toe)
○ 5 Tailbone (coccyx)
○ 6 Spine or back (vertebra)
○ 7 Upper arm or shoulder
○ 8 Elbow
○ 9 Lower arm or wrist
○ 10 Hand (not finger)
○ 11 Finger or toe
○ 12 Jaw, nose, face, and/or skull
○ 13 Ribs and/or chest or breast bone
○ 88 Another fracture not listed
Specify: ______________________

____

Final Instructions

Please take a moment to review any questions you may have missed. Feel free to write any comments in the Comments section below.

You may receive a follow-up call to clarify your answers on this form.

Please sign the enclosed Medical Record Release form and return both forms in the postage paid envelope.

Comments

Please report comments/additional provider information below. Provider information includes: Hospital/physician name, city and state, date of admission, length of stay, and reason for stay.

____

____

____

____

____

____

Thank you for completing this form!

PLEASE DO NOT WRITE IN THIS AREA

GPO U.S. GOVERNMENT PRINTING OFFICE:2020–776-900/40105
