# Supplementary material for: Women’s Health Initiative Strong and Healthy Pragmatic Physical Activity Intervention Trial for Cardiovascular Disease Prevention: Design and Baseline Characteristics
Source: J Gerontol A Biol Sci Med Sci. 2021 Jan 12;76(4):725–34. doi: 10.1093/gerona/glaa325 (PMC8011700; doi:10.1093/gerona/glaa325)
Supplement: glaa325_suppl_Supplementary_Appendix_B [file glaa325_suppl_supplementary_appendix_b.pdf]

WOMEN'S HEALTH INITIATIVE

Form 151– Activities of Daily Life

Ver. 9.2
OMB #0925-0414 Exp: 07/16

This form has questions about your current experiences. Please answer the questions as honestly as you can, using your first thoughts about each question. You should not go back later to "figure out" answers. Please answer the questions on both sides. Your answers will be kept confidential and will never be put with your name in a published report, but they will help us to understand the health of women like you. Thank you for your help.

1. In general, would you say your health is: **(Mark one circle only.)**

Excellent	Very good	Good	Fair	Poor
◯ 1	◯ 2	◯ 3	◯ 4	◯ 5

2. Overall, how would you rate your quality of life? **(Mark one circle below.)**

0	1	2	3	4	5	6	7	8	9	10
◯	◯	◯	◯	◯	◯	◯	◯	◯	◯	◯
Worst					Halfway					Best

As bad or worse than being dead — Best quality of life

3. Does the place (home, apartment, assisted living facility) where you live have special services for older people (such as help with transportation, meals, medicines, or bathing)?

◯ 0 No ◯ 1 Yes →

3.1. Are you currently receiving any of these services?
◯ 0 No ◯ 1 Yes

4. In the past year, have you stayed in a nursing home?

◯ 0 No ◯ 1 Yes

5. What aid, if any, do you usually use to walk on a level surface? **(Mark one.)**

I do not use any aid	I use a cane	I use crutches	I use a walker	I use a wheelchair
◯ 1	◯ 2	◯ 3	◯ 4	◯ 5

Public reporting burden for this collection of information is estimated to average 6 minutes per response, including the time for reviewing instructions, searching existing data sources, gathering and maintaining the data needed, and completing and reviewing the collection of information. An agency may not conduct or sponsor, and a person is not required to respond to, a collection of information unless it displays a currently valid OMB control number. Send comments regarding this burden estimate or any other aspect of this collection of information, including suggestions for reducing this burden, to: NIH, Project Clearance Branch, 6705 Rockledge Drive, MSC 7974, Bethesda, MD 20892-7974, ATTN: PRA (0925-0414). Do not return the completed form to this address.

AFFIX LABEL BETWEEN LINES
BAR CODE HERE

OFFICE USE ONLY

1. Date Received: Month - Day - Year

2. Reviewed By:

3. Contact Type:
◯ 1 Phone
◯ 2 Mail
◯ 8 Other ◯ FCA ◯ OU1 ◯ OU2

4. Language: ● 1 E ◯ 2 S

PLEASE MAKE NO MARKS IN THIS AREA

SERIAL #

GPO U.S. GOVERNMENT PRINTING OFFICE:2013–576-300/40077

Form 151– Activities of Daily Life

6. Are you taking a calcium supplement such as Oscal, Viactiv, or Tums?

   ○ 0 No　　○ 1 Yes

The following are questions about a typical (or usual) day's activities. Does your health now limit you in these activities and, if so, how much? (Mark one circle for each question.)

	No, not limited at all	Yes, limited a little	Yes, limited a lot
7. Vigorous activities, such as running, lifting heavy objects, or strenuous sports	○ 3	○ 2	○ 1
8. Moderate activities, such as moving a table, vacuuming, bowling, or golfing	○ 3	○ 2	○ 1
9. Lifting or carrying groceries	○ 3	○ 2	○ 1
10. Climbing several flights of stairs	○ 3	○ 2	○ 1
11. Climbing one flight of stairs	○ 3	○ 2	○ 1
12. Bending, kneeling, stooping	○ 3	○ 2	○ 1
13. Walking more than a mile	○ 3	○ 2	○ 1
14. Walking several blocks	○ 3	○ 2	○ 1
15. Walking one block	○ 3	○ 2	○ 1
16. Bathing or dressing yourself	○ 3	○ 2	○ 1

These next questions ask about how much help (if any) you need to do routine activities <u>for yourself</u>. Help can be defined as getting assistance from another person or using a device. (Mark one circle for each question.)

I can do this activity:	By myself without help	With some help	Completely unable to do this by myself
17. Can you feed yourself?	○ 1	○ 2	○ 3
18. Can you dress and undress yourself?	○ 1	○ 2	○ 3
19. Can you get in and out of bed yourself?	○ 1	○ 2	○ 3
20. Can you take a bath or shower?	○ 1	○ 2	○ 3
21. Can you do your own grocery shopping?	○ 1	○ 2	○ 3
22. Can you keep track of and take your medicines?	○ 1	○ 2	○ 3
