# Supplementary material for: Women’s Health Initiative Strong and Healthy Pragmatic Physical Activity Intervention Trial for Cardiovascular Disease Prevention: Design and Baseline Characteristics
Source: J Gerontol A Biol Sci Med Sci. 2021 Jan 12;76(4):725–34. doi: 10.1093/gerona/glaa325 (PMC8011700; doi:10.1093/gerona/glaa325)
Supplement: glaa325_suppl_Supplementary_Appendix_C [file glaa325_suppl_supplementary_appendix_c.pdf]

WOMEN'S HEALTH INITIATIVE

Form 521 - Physical Activity Questionnaire

Ver. 1

MARKING INSTRUCTIONS

- You may use a pencil or pen (blue or black only).
- To change an answer:
  - If you are using a pencil, cleanly erase the incorrect mark.
  - If you are using a pen, mark an X through the incorrect mark.
- Please do not make any stray marks on this form.

CORRECT MARK

INCORRECT MARKS

OFFICE USE ONLY

Date Received: ___ - ___ - ___
Month Day Year

○ FCA ○ OU1 ○ OU2

AFFIX LABEL HERE

These questions ask about physical activity and other habits that may affect your health. Please answer each question as accurately as possible. There are no right or wrong answers.

The first question is about your walking.

1. Think about the walking you do outside the home. How often do you walk outside the home for more than 10 minutes without stopping? **Mark only one.**

- ○ 0 Rarely or never → **Go to the next page.**
- ○ 1 1 to 3 times each month
- ○ 2 1 time each week
- ○ 3 2 to 3 times each week
- ○ 4 4 to 6 times each week
- ○ 5 7 or more times each week

(1–5) → **Go to Question 1.1 below.**

When you walk outside the home for more than 10 minutes without stopping,

1.1 For how many minutes do you usually walk?

- ○ 1 Less than 20 minutes
- ○ 2 20 to 39 minutes
- ○ 3 40 to 59 minutes
- ○ 4 1 hour or more

1.2 What is your usual speed?

- ○ 2 Casual strolling or walking (less than 2 miles an hour)
- ○ 3 Average or normal (2-3 miles an hour)
- ○ 4 Fairly fast (3-4 miles an hour)
- ○ 5 Very fast (more than 4 miles an hour)
- ○ 9 Don't know

00303

PLEASE MAKE NO MARKS IN THIS AREA

Form 521 - Physical Activity Questionnaire

2. When you exercise or walk in your usual fashion how would you rate your level of exertion (degree of effort)? **Mark only one.**

Nothing at all	Very, very weak (just noticeable)	Very weak	Weak	Moderate	Somewhat strong	Strong (heavy)	Very strong	Very, very strong (almost maximal)	Maximal
○ 0	○ 1	○ 2	○ 3	○ 4	○ 5	○ 6	○ 7	○ 8	○ 9

3. Are you able to walk at a normal pace for a half hour (30 minutes) or more?

○ 0 No ○ 1 Yes

4. Are you able to walk slowly for a half hour (30 minutes) or more?

○ 0 No ○ 1 Yes

5. During a usual day and night, about how many hours do you spend sitting? Be sure to include the time you spend sitting at work, sitting at the table eating, driving or riding in a car or bus, and sitting up watching TV or talking.

Less than 4 hours	4-5 hours	6-7 hours	8-9 hours	10-11 hours	12-13 hours	14-15 hours	16 or more hours
○ 1	○ 2	○ 3	○ 4	○ 5	○ 6	○ 7	○ 8

6. During a usual day and night, about how many hours do you spend sleeping or lying down with your feet up? Be sure to include the time you spend sleeping or trying to sleep at night, resting or napping, and lying down watching TV.

Less than 4 hours	4-5 hours	6-7 hours	8-9 hours	10-11 hours	12-13 hours	14-15 hours	16 or more hours
○ 1	○ 2	○ 3	○ 4	○ 5	○ 6	○ 7	○ 8

Go to the next page. →

7. On a typical **DAY**, how much time do you spend (from when you wake up until you go to bed) doing the following? **Mark only one answer per question.**

	None	15 min. or less	30 min.	1 hour	2 hours	3 hours	4 hours	5 hours	6 hours or more
7.1 Sitting while watching television (including videos on VCR/DVD).	○ 1	○ 2	○ 3	○ 4	○ 5	○ 6	○ 7	○ 10	○ 11
7.2 Sitting while using the computer for non-work activities or playing video games.	○ 1	○ 2	○ 3	○ 4	○ 5	○ 6	○ 7	○ 10	○ 11
7.3 Sitting while doing non-computer office work or paperwork not related to your job (paying bills, etc.).	○ 1	○ 2	○ 3	○ 4	○ 5	○ 6	○ 7	○ 10	○ 11
7.4 Sitting listening to music, reading a book or magazine, or doing arts and crafts.	○ 1	○ 2	○ 3	○ 4	○ 5	○ 6	○ 7	○ 10	○ 11
7.5 Sitting and talking on the phone or texting.	○ 1	○ 2	○ 3	○ 4	○ 5	○ 6	○ 7	○ 10	○ 11
7.6 Sitting in a car, bus, train, or other mode of transportation.	○ 1	○ 2	○ 3	○ 4	○ 5	○ 6	○ 7	○ 10	○ 11

Go to the next page. →

8. If you fell when you are away from your home, how confident are you that someone would be able to quickly help you?

- ○ 1 Not at all confident
- ○ 2 Somewhat confident
- ○ 3 Very confident
- ○ 9 Don't know/not sure

9. Have you had a fall in the **past 12 months**? By a "fall", we mean

- Fell all the way to the floor or the ground, or
- Fell and hit an object like a chair or stair

- ○ 0 No → **Go to page 6.**
- ○ 1 Yes ↓

10. How many times have you fallen in the **past 12 months**? (If you are unsure, make your best guess.)

- ○ 1 One time
- ○ 2 Two or three times
- ○ 3 Four or five times
- ○ 4 Six or more times

Go to the next page. →

11. At the time of your most recent fall, were you:

	No	Yes
11.1 Walking outside the home?	O 0	O 1
11.2 Doing strenuous exercise (but not walking outside the home)? Strenuous means you work up a sweat and would be exhausted by prolonged participation. For example, aerobics, aerobic dancing, jogging, tennis, swimming laps.	O 0	O 1
11.3 Doing moderate exercise (but not walking outside the home)? Moderate means exercise that is not exhausting. For example, biking outdoors, using an exercise machine (like a stationary bike or treadmill), calisthenics, easy swimming, popular and folk dancing.	O 0	O 1
11.4 Doing mild exercise? For example, slow dancing, bowling, or golf.	O 0	O 1
11.5 Doing other exercise (not previously listed)?	O 0	O 1
11.6 Doing strenuous indoor household chores (such as scrubbing floors, sweeping, or vacuuming)?	O 0	O 1
11.7 Working in the yard (such as mowing, raking, gardening, or shoveling snow)?	O 0	O 1

12. Were you injured as a result of any fall in the **past 12 months**?

O 0 No

O 1 Yes →

12.1 Please indicate what types of injuries. **Mark all that apply.**

O 1 Fracture

O 2 Laceration/cut

O 3 Bruising

O 4 Sprained or strained joint (wrist, knee, ankle, etc.)

O 5 Other injury (Please specify:______________________________)

12.2 Did you injure your head?

O 0 No

O 1 Yes

Go to the next page. →

Form 521 - Physical Activity Questionnaire

This set of questions is to help us understand the full range of activities you are doing. These questions are about activities that you may have done in the past 4 weeks. The questions on the following pages are similar to the example shown below.

If you **DID NOT** do the activity:

- Mark **NO** and move to the next question.

If you **DID** the activity in the past 4 weeks:

Step #1 Mark **YES**.

Step #2 Think about how many TIMES a week you usually did it and mark one answer.

Step #3 Mark one answer to show the TOTAL HOURS in a typical week you did the activity.

Here is an example of how Mrs. Jones would answer the first question: Mrs. Jones usually visits her friends Maria and Olga twice a week. She usually spends one hour on Monday with Maria and two hours on Wednesday with Olga. Therefore, the total hours a week that she visits with friends is 3 hours a week.

**EXAMPLE** **In a typical week during the past 4 weeks, did you…**	No	Yes	How many TIMES a week? Think in terms of days				How many TOTAL hours a week did you usually do it?					
			1	2	3-4	5 or more	Less than 1 hour	1-2.5 hours	3-4.5 hours	5-6.5 hours	7-8.5 hours	9 or more hours
Visit with friends or family (other than those you live with)?	○ 0	● 1	○ 1	● 2	○ 3	○ 4	○ 1	○ 2	● 3	○ 4	○ 5	○ 6

**13. In a typical week during the past 4 weeks, did you…**	No	Yes	How many TIMES a week? Think in terms of days				How many TOTAL hours a week did you usually do it?					
			1	2	3-4	5 or more	Less than 1 hour	1-2.5 hours	3-4.5 hours	5-6.5 hours	7-8.5 hours	9 or more hours
13.1 Jog or run (including use of treadmill)?	○ 0	○ 1	○ 1	○ 2	○ 3	○ 4	○ 1	○ 2	○ 3	○ 4	○ 5	○ 6

Go to the next page. →

PLEASE MAKE NO MARKS IN THIS AREA

00303

In a typical week during the past 4 weeks, did you...	No	Yes	How many TIMES a week? Think in terms of days				How many TOTAL hours a week did you usually do it?					
			1	2	3-4	5 or more	Less than 1 hour	1-2.5 hours	3-4.5 hours	5-6.5 hours	7-8.5 hours	9 or more hours
13.2 Walk uphill or hike uphill (count only uphill part; include use of treadmill)?	O 0	O 1	O 1	O 2	O 3	O 4	O 1	O 2	O 3	O 4	O 5	O 6
13.3 Walk fast or briskly for exercise (do not count walking leisurely or uphill; include use of treadmill)?	O 0	O 1	O 1	O 2	O 3	O 4	O 1	O 2	O 3	O 4	O 5	O 6
13.4 Walk to do errands (such as to/from a store or to take children to school (count walk time only)?	O 0	O 1	O 1	O 2	O 3	O 4	O 1	O 2	O 3	O 4	O 5	O 6
13.5 Walk leisurely for exercise or pleasure?	O 0	O 1	O 1	O 2	O 3	O 4	O 1	O 2	O 3	O 4	O 5	O 6
13.6 Ride a bicycle or stationary cycle?	O 0	O 1	O 1	O 2	O 3	O 4	O 1	O 2	O 3	O 4	O 5	O 6
13.7 Do other aerobic machines such as rowing, or step machines (do not count treadmill or stationary cycle)?	O 0	O 1	O 1	O 2	O 3	O 4	O 1	O 2	O 3	O 4	O 5	O 6
13.8 Do water exercises (do not count other swimming)?	O 0	O 1	O 1	O 2	O 3	O 4	O 1	O 2	O 3	O 4	O 5	O 6

Go to the next page. →

Form 521 - Physical Activity Questionnaire

**In a typical week during the past 4 weeks, did you…**	No	Yes	How many TIMES a week? Think in terms of days				How many TOTAL hours a week did you usually do it?					
			1	2	3-4	5 or more	Less than 1 hour	1-2.5 hours	3-4.5 hours	5-6.5 hours	7-8.5 hours	9 or more hours
13.9 Swim moderately or fast?	○0	○1	○1	○2	○3	○4	○1	○2	○3	○4	○5	○6
13.10 Swim gently?	○0	○1	○1	○2	○3	○4	○1	○2	○3	○4	○5	○6
13.11 Do stretching or flexibility exercises (do not count yoga or Tai-chi)?	○0	○1	○1	○2	○3	○4	○1	○2	○3	○4	○5	○6
13.12 Do yoga or Tai-chi?	○0	○1	○1	○2	○3	○4	○1	○2	○3	○4	○5	○6
13.13 Do aerobics or aerobic dancing?	○0	○1	○1	○2	○3	○4	○1	○2	○3	○4	○5	○6
13.14 Do moderate to heavy strength training (such as hand-held weights of more than 5 lbs., weight machines, or push-ups)?	○0	○1	○1	○2	○3	○4	○1	○2	○3	○4	○5	○6
13.15 Do light strength training (such as hand-held weights of 5 lbs. or less or elastic bands)?	○0	○1	○1	○2	○3	○4	○1	○2	○3	○4	○5	○6
13.16 Do general conditioning exercises, such as light calisthenics or chair exercises (do not count strength training)?	○0	○1	○1	○2	○3	○4	○1	○2	○3	○4	○5	○6

Thank you for completing this questionnaire.
Please take a moment to review any questions you may have missed.

PLEASE MAKE NO MARKS IN THIS AREA

00303
