# Supplementary material for: Women’s Health Initiative Strong and Healthy Pragmatic Physical Activity Intervention Trial for Cardiovascular Disease Prevention: Design and Baseline Characteristics
Source: J Gerontol A Biol Sci Med Sci. 2021 Jan 12;76(4):725–34. doi: 10.1093/gerona/glaa325 (PMC8011700; doi:10.1093/gerona/glaa325)
Supplement: glaa325_suppl_Supplementary_Appendix_D [file glaa325_suppl_supplementary_appendix_d.docx]

**Appendix D: *WHISH* Accelerometry Substudy**

**Sample selection**

A subcohort of WHISH participants were invited to join the “WHI Physical Activity Follow-up Study” to provide objective data on changes in physical activity and time spent sedentary (measured by accelerometers). These women were part of a previous WHI ancillary study of 6,489 participants who in 2012-2014 participated in the Objective Physical Activity and Cardiovascular Health (OPACH) Study (R01 HL105065, AZ LaCroix, PI). Details on the design and implementation of the OPACH Study have been published previously.^1^ Briefly, all women were ambulatory, community-living, and without major cognitive decline at OPACH baseline. African American and Hispanic women were oversampled and comprised 50% of OPACH participants—the average age was 79±7 years. Surviving members of the OPACH cohort who had successfully worn and returned accelerometers were mailed invitations and consent forms beginning in February 2015. This subcohort was targeted for device-monitored changes in physical activity because of they had previous experience using accelerometers and because the OPACH measurement provided a pre-WHISH Trial measurement for comparison. To achieve balanced groups of at least 1,000 women in both of the WHISH study groups throughout the planned follow-up period, the recruitment target was set at 2,300 and 2,349 women consented.

**Accelerometer deployment and wear protocol**

Accelerometer data collection was conducted by the Exercise and Physical Activity Resource Center at UC San Diego <http://ucsdeparc.ucsd.edu>. Participants who consented were mailed a package containing an Actigraph GT3X+ accelerometer, an elastic waist band, a sleep journal to track in-bed and out-of-bed times, and instructions for wearing and returning the accelerometer in an addressed express mail return envelope. Women were instructed to wear the accelerometer over the right hip, 24-hours per day for 7 days, and to remove them only for bathing and other water-based activities. If women were unwilling to wear the accelerometer at night, they were instructed to remove it when they got into bed, and to replace it upon getting out of bed. Participants were given a phone number to call for assistance during the accelerometer wear period, and at least 1 phone call was made to each participant during accelerometer wear to facilitate accurate placement and adherence. Participants were sent a thank-you letter and a $10 gift card upon receipt of their accelerometer and sleep logs. Accelerometers were deployed at three separate time points–at 6, 18, and at 36 months, so that along with the OPACH baseline, up to four separate weeks of accelerometer-measured physical activity is available on this subcohort of WHISH participants. Of the 2,349 women who consented to the *WHISH* Accelerometry Substudy, 85.4% returned an accelerometer in Year 1 of *WHISH*. Of the women who were still alive, 75.1% returned accelerometers in Year 2*,* and 67.3% returned accelerometers in Year 4.

**Data processing (including variables)**

As accelerometers were returned by mail, raw data were stored on a secure server and ActiLife v6 was used to integrate data to 15-second epochs using the normal filter and the low-frequency filter. Raw data were also converted to 41 descriptive features for each 1-minute epoch—the resulting features were input to a random forest machine learning (ML) classifier that was developed and validated specifically for older women in a separate study^2^ to measure sitting, standing without ambulation, standing with ambulation (i.e., puttering), and walking/running. In-bed time was identified in the 15-second epoch files and the 1-minute ML classified files using data from sleep journals, with missing data imputed using person-specific times if available, and the overall sample in-bed and out-of-bed times if person-specific times were not available. Non-wear time was identified using the commonly used^3^ Choi algorithm^4,5^ applied to the vector magnitude of counts-per-minute that identified 90 minute intervals with 0 counts per minute as non-wear time, requiring a 30-minute stream frame and allowing for one 2-minute tolerance. Each 15-second epoch of awake wear time was classified as either sedentary, light physical activity (PA), or moderate-to-vigorous PA (MVPA) using vector magnitude cutpoints determined in the OPACH laboratory-based calibration study of 200 older women using the respective counts per 15-second cutpoints of ≤18, 19 through 518, and ≥ 519.^6^ Days with 10 or more hours of awake wear time were considered adherent,^3^ and sedentary time, light PA, MVPA, sitting time, standing without ambulation, standing with ambulation, and walking/running were computed by averaging the respective minutes spent in each activity over all adherent days for each participant.

REFERENCES

1. LaCroix AZ, Rillamas-Sun E, Buchner D, et al. The Objective Physical Activity and Cardiovascular Disease Health in Older Women (OPACH) Study. *BMC Public Health*. 2017;17(192). doi:10.1186/s12889-017-4065-6

2. Rosenberg D, Godbole S, Ellis K, et al. Classifiers for Accelerometer-Measured Behaviors in Older Women. *Med Sci Sports Exerc*. 2017;49(3):610-616. doi:10.1249/MSS.0000000000001121

3. Migueles JH, Cadenas-Sanchez C, Ekelund U, et al. Accelerometer Data Collection and Processing Criteria to Assess Physical Activity and Other Outcomes: A Systematic Review and Practical Considerations. *Sport Med*. 2017;47(9):1821-1845. doi:10.1007/s40279-017-0716-0

4. Choi L, Liu Z, Matthews CE, Buchowski MS. Validation of accelerometer wear and nonwear time classification algorithm. *Med Sci Sports Exerc*. 2011;43(2):357-364. doi:10.1249/MSS.0b013e3181ed61a3.Validation

5. Choi L, Ward SC, Schnelle JF, Buchowski MS. Assessment of wear/nonwear time classification algorithms for triaxial accelerometer. *Med Sci Sports Exerc*. 2012;44(10):2009-2016. doi:10.1249/MSS.0b013e318258cb36

6. Evenson KR, Wen F, Herring AH. Associations of Accelerometry-Assessed and Self-Reported Physical Activity and Sedentary Behavior with All-Cause and Cardiovascular Mortality among US Adults. *Am J Epidemiol*. 2016;184(9):621-632. doi:10.1093/aje/kww070
